# Neutrophil and macrophage apoptosis in bronchoalveolar lavage fluid from healthy horses and horses with recurrent airway obstruction (RAO)

**DOI:** 10.1186/1746-6148-10-29

**Published:** 2014-01-24

**Authors:** Artur Niedzwiedz, Zbigniew Jaworski, Bartlomiej Tykalowski, Marcin Smialek

**Affiliations:** 1 Department of Internal Diseases with Clinic for Horses, Dogs and Cats, The Faculty of Veterinary Medicine, Wrocław University of Environmental and Life Sciences, Pl. Grunwaldzki 47, Wrocław 50-366, Poland; 2 Department of Horse Breeding and Riding, University of Warmia and Mazury, Prawocheńskiego 2, Olsztyn 10-720, Poland; 3 Department of Poultry Diseases, University of Warmia and Mazury, Oczapowskiego 13, Olsztyn 10-719, Poland

## Abstract

**Background:**

Dysregulation of apoptosis has been implicated in a range of diseases including tumors, neurodegenerative and autoimmine diseases, as well as allergic asthma and chronic obstructive pulmonary disease (COPD) in humans. Although it has a different pathophysiology, delayed apoptosis of various inflammatory cells may play a pivotal role in the development of recurrent airway obstruction (RAO) in horses. Reduction of inflammatory cell apoptosis or a dysregulation of this process could lead to chronic inflammation and tissue injury. Therefore, the aim of this study was to investigate the rate of apoptosis and necrosis of neutrophils and macrophages in bronchoalveolar lavage fluid obtained from seven horses suffering from RAO (study group) and seven control horses.

**Results:**

We demonstrated that neutrophil/macrophage apoptosis is altered in RAO-affected horses compared with the control group in the BAL fluid. We found a significant difference between the median percentage of early and late apoptosis of neutrophils between the study and control group of horses. Moreover, we found a positive correlation between the rate of apoptosis and the median percentage of macrophages in RAO-affected horses.

**Conclusion:**

The findings suggest that apoptosis dysregulation may play a significant role in the pathogenesis of RAO. However, further studies are needed to clarify the role of altered apoptosis in the course of equine recurrent airway obstruction.

## Background

Recurrent airway obstruction (RAO, “heaves”), also known as ‘broken wind’, is an asthma-like condition that develops in mature horses following stabling and exposure to dusty hay and straw, affecting a large number of animals worldwide. Affected horses develop airway bronchoconstriction, neutrophilic inflammation and airway hyper-responsiveness. A number of environmental, immunological, infectious and genetic factors play an important role in the pathogenesis of RAO. However, the immunological basis of the disease is still not fully understood. The immediate-type I and the immune complex formation type III hypersensitivity reactions play a pivotal role in the development of airway inflammation. The most common clinical signs are cough exacerbated after exercise or in dusty environments, nasal discharge, exercise intolerance and respiratory difficulty [1]. Pathological changes in the airways are present as a result of neutrophilic inflammation, a mucous hypersecretion and bronchospasm [2,3]. The degree of airway obstruction may vary depending on the stage of the disease and can be reversed either by medication or a change of the environment. Clinical signs as well as airway inflammation wane if RAO-affected horses remain pastured for a prolonged time. Once the animals are returned to the stable and dusty environment, breathing problems relapse [4].

During an exacerbation of the disease in the in RAO-affected horses, the cytological evaluation of the bronchoalveolar lavage fluid shows a higher proportion of nondegenerated segmented neutrophils (usually more than 20%, in severe cases approximately 60-85%) compared with that obtained from healthy horses, which contains approximately 60% macrophages, 35% lymphocytes, <5% neutrophils and <2% mast cells [5].

Apoptosis is the process of programmed cell death and is a highly ordered physiological process by which irreversibly damaged or useless cells are eliminated throughout one’s entire life. It consists of highly coordinated molecular events leading to a sequence of morphological changes and is accompanied by modifications of the cellular surface. The cell loses its surface anti-phagocytic signals and exposes ligands, such as phosphatidylserine, designating the cell for phagocytosis. Importantly, the early apoptotic cells preserve their plasma membrane integrity to retain the potentially harmful cellular contents inside. If not successfully taken up by phagocytes, apoptotic cells proceed to the phase of late apoptosis (also termed secondary necrosis), where the plasma membrane becomes permeable to small molecules (e.g. propidium iodide (PI)) and, subsequently, also macromolecules (proteins). Dysregulation of apoptosis has been implicated in a range of diseases including tumors, neurodegenerative and autoimmune disorders, as well as allergic asthma and COPD in humans [6-8]. This process has attracted great attention in horses in recent years, and several studies concerning apoptosis of different lymphocyte subpopulations in RAO-affected horses have been conducted, since delays in the apoptotic response may be associated with persistent inflammation and subsequent tissue damage [9-11]. However, to the authors’ knowledge, only limited research is available concerning the role of cell types other than lymphocytes in RAO affected horses [10,12]. Using fluorescence microscopy, Breuer et al. found a significant increase in the percentage of all necrotic cells in bronchoalveolar lavage fluid (BALF) obtained from RAO-affected horses compared to healthy controls. However, no difference was found between the percentage of apoptotic and viable BALF cells. In this study, the authors also used flow cytometry to compare the percentage of necrotic, apoptotic and viable cells with respect to cells with high granularity, macrophages and lymphocytes. However, due to relatively small groups of horses, the authors did not conduct any statistical examination of the results [10]. A study by Turlej et al. showed a significant delay in the apoptosis of granulocytes obtained during bronchoalveolar lavage in horses with heaves compared to healthy horses, while the level of apoptosis of granulocytes, obtained from the peripheral blood, was comparable between the two groups [12]. Additional knowledge about the regulating mechanisms of apoptosis in all lung cells would contribute to the better understanding of the pathophysiology of equine lung disorders and allow the development of a targeted therapy.

Having hypothesized that the accumulation of neutrophils and macrophages in the airways of RAO-affected horses can result from decreased apoptosis, the present study was performed to quantify and characterize the apoptosis of airway neutrophils/macrophages recovered by bronchoalveolar lavage from RAO-affected and healthy horses.

## Methods

This study was conducted with the approval of the 2nd Local Ethics Committee concerning Animal Experimentation in Wrocław (resolution No 1/2012).

### Horses

Fourteen adult Polish Konik horses kept in uniform environmental and living conditions were used in this study. Horses were qualified into the groups based on their history. 7 horses were not affected by RAO (control horses) and 7 horses had a history of RAO (RAO-affected horses). All horses were owned by the Polish Academy of Sciences Research Station for the Ecological Agriculture and Preservation of Animal Breeding in Popielno (latitude 53˚75′ N, longitude 21˚62′ E). The control group consisted of five castrated males (geldings) and two mares, whose ages ranged from 5-13 years (mean ± SD, 7.7 ± 2.9), their body weights ranged from 350 to 400 kg and they measured approximately 110–130 cm at the withers). The study group consisted of four geldings and three mares. Their ages ranged from 7-14 years (mean ± SD, 9.8 ± 2.6), their body weights varied between 350 and 400 kg, they measured approximately. 110–130 cm at the withers. and had a history of chronic respiratory disease following exposure to moldy hay and/or a dusty environment (RAO).

### Experimental protocol

Prior to the study, all horses were kept on a pasture or in a stable with wood shavings for a minimum of 8 weeks to ensure the RAO-affected horses were in complete clinical remission. An acute crisis of heaves in suitable animals was induced by placing all horses in a poorly ventilated stable, bedding them on straw and feeding them hay with visible mold growth for 48 hours prior to examination. Hay and straw were not shaken up additionally, since the introduction of the horses into the poorly ventilated stable has been shown to suffice in inducing airway inflammation and RAO exacerbation in selected horses [11]. An inflammatory response to the poor environmental conditions was confirmed using a clinical RAO score, an endoscopic examination and bronchoalveolar lavage fluid (BALF) cytology, arterial blood gas analysis and venous blood samplings [13,14].

### Clinical evaluation

Horses underwent only one full clinical examination after a period of 48 h of dust exposure, including a visual inspection, measurement of the internal rectal body temperature, pulse and respiratory rate, evaluation of the appearance of mucous membranes, auscultation of the heart, intestinal peristalsis and recording of the capillary refill time (CRT). The clinical RAO score, assessing the condition of the respiratory system based on a cough score, nostril flare and abdominal lift, was performed according to guidelines previously described by Robinson et al. [15]. Venous blood was collected from the external jugular vein for hematology, screening biochemistry and the analysis of acute phase proteins [14]. Samples were maintained on ice immediately after collection and analyzed within 6 hours.

### Arterial blood gas analysis

Arterial blood was collected anaerobically into heparinized syringes through an arterial puncture of the facial artery using an 18G butterfly needle. The blood was immediately analyzed for the partial pressure of oxygen and carbon dioxide (PaO_2_ and PaCO_2_) with the use of an OPTI CCA-TS (OPTI Medical Systems, Inc., Roswell, GA, USA) blood gas analyzer. Horses were identified as healthy, if their PaO2 ≥ 90 mmHg, while horses with a relapse of RAO had to have a PaO2 ≤ 85 mmHg [16,17].

### Endoscopic examination, bronchoalveolar lavage fluid collection and cytology

Horses were sedated with 0.01 mg/kg of detomidine (Domosedan®, Orion Corporation, Finland) and 0.01 mg/kg of butorphanol (Butomidor®, Richter Pharma AG, Austria) prior to an endoscopy of the airways and a bronchoalveolar lavage. A twitch had to be additionally used in some horses. A 1.8 m long endoscope (Karl Storz) was then passed through the nasal passage and trachea until it was wedged in the right dorsocaudal lung lobe. Videos and pictures were recorded and changes in the airways were graded by two clinicians according to the quantity and quality of the mucus, using a modified RAO staging scale previously described by Tilley et al. [18] (see Additional file 1: Table S1). Bronchoalveolar lavage was performed by instilling 250 ml of sterile saline (0.9% NaCl) at body temperature through the endoscope working channel into a bronchus using successive 60 ml boluses, and reaspirating BALF through gentle suction using a 60 ml syringe until no further fluid was obtained [19]. The amount of reaspirated fluid was recorded and BALF was pooled in a sterile specimen cup without any medium, placed on ice and processed within 2 hours after collection for each individual horse.

10 ml sample aliquots were cytospinned at 300 × *g* for 10 min using the Beckman Coulter Allegra ×-22 cytospin and stained with Wright’s stain in order to make smears. A 400-cell leukocyte differential count (×1000 magnification) was performed wherein epithelial cells were not taken into account [20].

### Staining for the evaluation of apoptosis

The Southern Biotech (Birmingham, AL 35209, USA) ApoScreen™ Annexin V Apoptosis Kit was used. Prior to staining, cells were concentrated to 1 × 10^6^ to 1 × 10^7^ cells/ml. A Vi-Cell XR (Beckman Coulter) was used to assess the cell concentration. Cells were washed in cold PBS twice, after which PBS was removed from the cell pellet. Next, the cells were washed in 1 mL of 1× Annexin V binding buffer once. The supernatants were removed by centrifugation and the cells were suspended in 1× Annexin V binding buffer. 10 μL of FITC-conjugated Annexin V and 10 μL of propidium iodide (PI) were added to the cells. They were then mixed gently and incubated for 15 min on ice in the dark, then diluted with 380 μL of 1× Annexin V binding buffer and analyzed by flow cytometry within 1 h [21,22].

### FACS acquisition and analysis

**Figure 1:**
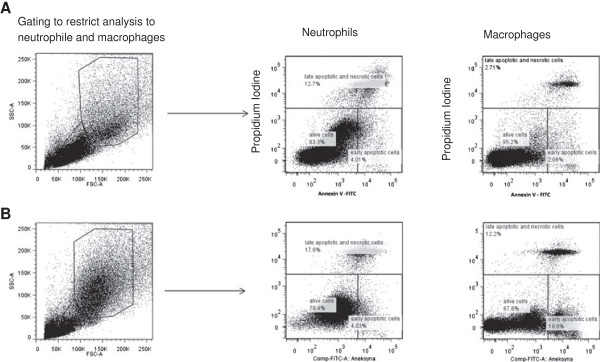
**Examples of dot plot cytograms showing percentages of early apoptotic (Annexin V+PI-), late apoptotic/necrotic (Annexin V+PI+) and viable (Annexin V-PI-) neutrophils/macrophages in normal control (A) and RAO-affected horses (B).** Left pictures shows gating process to obtain only neutrophile and macrophages region. Lymphocytes and epithelial cells were excluded from gating.

Flow cytometry analysis was performed using a FACSCanto II cytometer (BD Biosciences). The data were acquired using version 6.1.3 of the FACSDiva software (BD Biosciences) and analyzed using FlowJo software (Tree Star Inc., Stanford, CA, USA). The cytometry setup and tracking beads (CST, BD Biosciences) were used to initialize PMT settings. Unstained control cells and a single stain control for each fluorochrome were prepared and used to set up flow cytometric compensation. Between 15,000 and 20,000 gated cells were analyzed. Apoptosis was assessed in the total macrophage and neutrophil population by the surface exposures of plasma membrane phosphatidyl serine (Annexin V+cells) and loss of plasma membrane integrity (PI+cells). A macrophage and neutrophil region were established according to their different light-scattering properties: forward scatter (FSC) which reflects cell size, and side scatter (SSC), which depends on the intensity and diffraction capacity of the cells (complexity/granularity) [23,24]. The dot plot of Annexin V versus PI was used for the assessment of apoptosis. Annexin V-/PI- cells were considered viable; Annexin V+/PI- cells were deemed early-apoptotic; and double positive cells (Annexin V+/PI+) were considered to be a mixture of late apoptotic and primary necrotic cells (Figure 1).

### Statistical analysis

Analysis using the Anderson Darling test indicated that data were not distributed normally. The median, 25th and 75th percentiles are presented. The nonparametric U Mann-Whitney test was performed to evaluate the results. This analysis was performed using the STATISTICA v. 10.0 software (StatSoft, Tulsa, OK, USA). P < 0.05 was considered as statistically significant.

## Results

### Bronchoalveolar lavage cytology and clinical score

The bronchoalveolar lavage fluid cytology results, clinical assessment and blood gas analysis are summarized in Table 1. Based on these findings and the history of each horse, control horses were considered healthy and respiratory diseases free when they obtained a total clinical RAO score of less than 1, and their BALF cytology count showed less than 10% of neutrophils. Although reference ranges of the percentage of neutrophils in BALF of healthy horses are <5%, we considered the neutrophil count of <10% as a normal result, since stabling in a dusty environment has been shown to increase the neutrophil percentage in the BALF of healthy horses [25]. However, the median percentage of neutrophils in the control group was 5.1%. Horses from the RAO-affected group had a history of episodes of RAO and their total clinical RAO score was greater than or equal to 5, and they had more than 20% neutrophils in their BALF cytology. The mucus scores were generally low in the control horses and, apart from mucus accumulation, all median variables in this group were below 1. Only two individuals from this group had a mucous score of 2, while the remaining horses had a very small amount of mucus or none at all. There was a statistically significant difference (p < 0.05) between all endoscopic examination variables, when comparing the control and study group. In the study group, all median variables were above 2 and above 3 with regards to mucus viscosity.

**Table 1:** Results of BAL fluid cytology, clinical assessment and blood gas analysis in healthy horses and RAO-affected horses

	**Healthy **_**n=7**_	**RAO-affected **_**n=7**_
*Clinical score*^*a*^	2.0 (2 and 2)	6 (5 and 6)
*PaO2 (mmHg)*^*a*^	91 (85 and 107)	96 (91 and 108)
*PaCO2 (mmHg)*^*b*^	45 (43 and 46)	45.5 (44 and 50)
*BALF neutrophils (%)*^*a*^	5.1 (4.1 and 5.3)	59.8 (51.3 and 64.8)
*BALF lymphocytes (%)*^*a*^	41 (38.5 and 45.9)	38.1 (34.8 and 41.1)
*BALF macrophages (%)*^*a*^	55.8 (49.8 and 59.1)	32.8 (25.9 and 35.7)
*BALF eosinophils (%)*^*a*^	0.4 (0.2 and 0.5)	0 (0 and 0)
*BALF mast cells (%)*^*b*^	0.1 (0 and 0.3)	0 (0 and 0)

Values are expressed as median and 25th and 75th percentiles.

^a^Differences statistically significant (P < 0.05).

^b^NS.

The average BALF reaspiration was significantly different (p < 0.001) between both groups and was 56.6% (56.6 ± 9.9) in the control horses, and 43.6% (43.6 ± 12.6) in the RAO-affected horses. The median and ranges of the BALF differential cell count in the control horses were as follows: 56.4 ± 9.5 macrophages, 42.1 ± 8.9 lymphocytes and 4.8 ± 2.3 neutrophils. In the RAO-affected horses, they amounted to 28.4 ± 11.2 macrophages, 38.9 ± 12.4 lymphocytes and 58.9 ± 7.9 neutrophils. The mean percentage of neutrophils was significantly higher in the group of RAO-affected horses when compared to the healthy control group (p < 0.001), whereas the mean percentage of macrophages was significantly higher in the control group compared to the RAO-affected horses (p < 0.001). The percentage of lymphocytes in the group of healthy horses was also significantly higher compared to the group of RAO-affected horses (p < 0.05).

### Neutrophil and macrophage apoptosis

The summarized results of the apoptosis of neutrophils and macrophages are shown in Table 2. There was a significant difference (p < 0.05) between the median percentage of viable neutrophils obtained from the control group (median = 85.9%, 25th and 75th percentiles = 76.7 and 92.2) and that from the group of the RAO-affected horses (median = 89.5%, 25th and 75th percentiles = 78.9 and 91.3). The BALF neutrophils obtained from the RAO-affected horses were less AnnexinV+/PI- (early apoptotic) than those from the control subjects (median 5.19% 25th and 75th percentiles, 3.48 and 6.33 *versus* 12.3% 25th and 75th percentiles 4.69 and 19.8, respectively, p < 0.05). The median percentage of AnnexinV+/PI+(late apoptotic) neutrophils was higher in the control group than in the RAO-affected horses (4.7%, 25th and 75th percentiles, 2.96 and 3.94 and 4.09%, 25th and 75th percentiles 3.88 and 6.4, respectively, p < 0.05), as shown in Table 2.

**Table 2:** The results are expressed as a percentage of viable, early apoptotic and late apoptotic/necrotic cells within macrophage and neutrophils populations

	**Viable neutrophils**^**a**^	**Eearly apoptotic neutrophils**^**a**^	**Late apoptotic neutrophils**^**a**^	**Viable macrophages**^**b**^	**Early apoptotic macrophages**^**a**^	**Late apoptotic macrophages**^**a**^
**Healthy**	85.9 (76.7 and 92.2)	12.3 (4.69 and 19.8)	4.7 (2.96 and 3.94)	83.0 (78.7 and 92.3)	3.53 (3.5 and 4.89)	10.8 (4.14 and 17.6)
**RAO-affected**	89.5 (78.9 and 91.3)	5.19 (3.48 and 6.33)	4.09 (3.88 and 6.4)	81.1 (71.5 and 85.7)	4.2 (3.51 and 6.09)	12.7 (8.62 and 23.6)

Values are expressed as median and 25th and 75th percentiles.

^a^Differences statistically significant (P < 0.05).

^b^NS.

The percentage of viable macrophages from the healthy controls (median = 83%, 25th and 75th percentiles, 78.7 and 92.3) was higher compared to those from RAO-affected individuals (median = 81.1%, 25th and 75th percentiles, 71.5 and 85.7), however no statistical differences were observed. In contrast to neutrophils, the median BALF macrophages from RAO-affected horses were more AnnexinV+/PI- than those from the healthy controls (4.2%, 25th and 75th percentiles,3.51 and 6.09 *versus* 3.53% 25th and 75th percentiles, 3.5 and 4.89 respectively, p < 0.05). Moreover, the median percentage of AnnexinV+/PI+macrophages was higher in the group of RAO-affected horses compared to healthy controls (12.7% 25th and 75th percentiles, 8.82 and 23.6and 10.8% 25th and 75th percentiles, 4.14 and 17.6, respectively, p < 0.05).

## Discussion

In our study, two groups of horses were evaluated and compared with regard to several variables of the respiratory system. The experimental protocol included sample collection and the examination of results acquired from healthy Polish Konik horses, and those suffering from RAO. The results obtained in the study group (almost 60% of nondegenerated neutrophils) clearly demonstrate RAO as a direct cause of a chronic respiratory problem. It is interesting to note that the results obtained from the control group are slightly above the reference ranges, despite the fact that these horses were stabled in a dusty barn for 48 h and fed poor quality, moldy hay. Our data are inconsistent with results reported by Holcombe et al. (2001), who showed that stabling is associated with airway inflammation and significant changes in the BAL cytological profile [25]. The most likely explanation of the slightly elevated percentage of neutrophils in the control group in this study is that it was a response to the stabling conditions. However, since baseline BAL results of the horses prior to stabling were not obtained, this is only an assumption.

Several mechanisms contribute to the pathogenesis of RAO in horses. First, inhalation of molds, dust or noxious particles such as ammonium, cause the migration and presence of inflammatory cells in the bronchi, leading to chronic inflammation [1]. Secondly, there is a disruption in mucous cell hyperplasia in RAO-affected horses [12]. The third mechanism in the pathogenesis of RAO involves oxidative stress and disturbances between antioxidants and free radicals [26]. There may be a fourth hypothetical mechanism involved in the pathogenesis of the disease in horses, involving a disruption of the balance between apoptosis and necrosis of the inflammatory cells that infiltrate equine airways [10,11].Inflammation and a persistent presence of granulocytes and other leukocytes in the lower airways can be explained an increased neutrophil activation and chemotaxis [12,27] and a delayed tissue clearance due to delayed apoptosis or dysfunction in the clearance of these cells. It has been proven that delayed apoptosis is associated with inflammatory diseases such as cystic fibrosis and pneumonia and different types of neoplasm [6-8]. Moreover, similar findings have been demonstrated in human asthma, where delayed apoptosis was seen in peripheral blood eosinophils [6,28].

In the present study, we demonstrated that neutrophil/macrophage apoptosis is altered in RAO-affected horses compared with the control group in BAL fluid. The median percentage of viable neutrophils was significantly higher in the RAO affected horses than in the controls, suggesting that a higher percentage of neutrophils can result from specific survival factors at the site of inflammation. Previous studies have also demonstrated delayed apoptosis of BALF neutrophils. In a study by Turlej et al. BALF neutrophils from RAO-affected horses demonstrated a significant delay in apoptosis together with blood neutrophils from the sick and healthy horses, in contrast to BALF neutrophils of control animals [12]. The rate of peripheral blood neutrophil apoptosis was comparable, suggesting that an enhanced survival of BALF neutrophils is the result of the action of specific cytokines, which affect granulocytes that have extravasated into the inflamed lungs. A key contributing factor in delayed apoptosis seems to be the granulocyte/macrophage colony stimulating factor (GM-CSF) [29-31]. The local concentration of GM-CSF is increased in airway diseases characterized by an accumulation of neutrophils, such as RAO in horses or, asthma in humans. It has been shown that GM-CSF delays equine neutrophil apoptosis by activating the STAT3 and STAT5 signal transducer and activator of the (STAT) transcription family members, which are critical regulators of the expression of various Bcl-2 family proteins [12,29]. As mentioned in the introduction, Breuer et al. did not conduct a statistical analysis with respect to specific leukocyte subpopulations. However there are appear to be relatively high values between apoptotic and viable cells with high granularity in the RAO-affected horses. Despite the lack of a statistical comparison, it seems that results of our study are comparable.

In our study, we found a significant difference between the median percentage of early and late apoptosis occurring within neutrophils in the study and control group of horses. A higher proportion of early and late apoptotic neutrophils can explain a lower number of these cells in general in the lungs of healthy horses. Large numbers of apoptotic cells are constantly generated *in vivo* and phagocytes such as macrophages should remove apoptotic cells as soon as they appear. However, it should be noted that late apoptotic cells are phagocytized much more significantly than early apoptotic cells, which can explain the large difference in median percentages between early and late apoptotic neutrophils in healthy horses.

Finally, in our study, we found a positive correlation between the rate of apoptosis and median percentage of macrophages in the RAO-affected horses. . While there are two reports on the rate of apoptosis of neutrophils, to the authors’ knowledge there is only one study evaluating the apoptosis of alveolar macrophages. However, it lacks a statistical assessment [10,12]. Taking into consideration the results of this study, our observations are similar with the numbers and medians of Breuer et al. except the necrotic macrophages, [10]. Alveolar macrophages are an important source of cytokines, which can participate in tissue damage. However, cytokines can also influence the apoptotic death of macrophages: macrophage apoptosis can be increased by interferon-gamma and reduced by IL-4, IL-10 and TGF-b [32]. Moreover, an increase in the early and late rate of apoptosis in macrophages from RAO-affected horses can explain a lower percentage of these cells in BALF obtained from these animals compared with healthy controls.

This study has some limitations. Firstly, the sample size is small. Although differences between the groups are statistically significant, the data should be confirmed by further controlled studies engaging a larger population of horses. Secondly, we did not use special staining methods to evaluate macrophages and neutrophils. Such staining could help to better classify the cells.

## Conclusion

In conclusion, the findings suggest that pulmonary neutrophil/macrophage apoptosis is altered in RAO in horses. This disturbance plays an important role in the pathogenesis of allergic diseases both in humans and in horses. A detailed study on the signaling pathway in lung granulocytes/macrophages in RAO-affected horses can lead to a new therapeutic approach to this disease. Therefore, more studies are needed to investigate apoptosis and its control mechanism in the course of RAO in horses.

## Competing interests

None of the authors of this paper have a financial or personal relationship with other people or organizations that could inappropriately influence or bias the content of the paper.

## Authors’ contributions

AN designed the study, conducted the experiments, collected all samples, performed the blood and BALF analysis. ZJ was responsible for the animals, helped obtain the samples and write the manuscript. BT and MS performed the FACS analysis. All authors read and approved the final manuscript.

## Supplementary Material

Additional file 1: Table S1 Modified clinical staging of RAO in horses according to Tilley et al. [18]. Click here for file
