# Supplementary material for: Neutrophil and macrophage apoptosis in bronchoalveolar lavage fluid from healthy horses and horses with recurrent airway obstruction (RAO)
Source: BMC Vet Res. 2014 Jan 24;10:29. doi: 10.1186/1746-6148-10-29 (PMC3903020; doi:10.1186/1746-6148-10-29)
Supplement: Additional file 1: Table S1 — Modified clinical staging of RAO in horses according to Tilley et al. [18]. [file 1746-6148-10-29-S1.pdf]

Additional file 1: Table S1

Modified clinical staging of RAO in horses according to Tilley et al [18]

Parameter	**0**	**1**	**2**	**3**	**4**	**5**
**Clinical assessment**[a]						
Cough score	None	Coughs on specific times of day (feeding/ exercising/making beds)	Frequent cough with periods of no coughing	Very frequent cough		
Nostril flare	None	Flares during inspiration (returns to normal at end inspiration)	Flares in inspiration and exhalation (slight movement can still be seen)	Flares in inspiration and expiration (no movement can be seen)		
Abdominal lift	None	Slight flattening of ventral flank	Obvious abdominal flattening and ‘‘heave line’’ extending no more than half way between cubital joint and *tuber coxae*	Obvious abdominal lift and ’’heave line’’ extending beyond halfway between cubital joint and *tuber coxae*		
**Airway endoscopy**[b]						
Mucus accumulation	None, clean	Little, multiple small blobs	Moderate, larger blobs	Marked, confluent or stream-forming	Large, pool-forming	Extreme, profuse amounts
Mucus color	None, clean	Colorless	White	Thick White	Yellow	Thick Yellow
Mucus localization and stickiness	None, clean	1/2 Ventral	2/3 Lateral	3/4 Dorsal	Threading	Threading
Mucus apparent viscosity	None, clean	Very fluid	Fluid	Intermediate	Viscous	Very viscous

[a] Final **C**linical **S**core (CS): **0** (CS final score < 2), **1** (2 ≤ CS final score ≤ 4) **2** (5 ≤ CS final score ≤ 6) **3** (7 ≤ CS final score ≤ 9)

[b] Final airway **E**ndoscopy **S**core: **0** (ES final score < 8.5) **1** (8.5 ≤ ES final score ≤ 12) **2** (12 < ES final score ≤ 16) **3** (ES final score > 16)

Final RAO Stage:

Stage 0 – No RAO (Total Score = 0);

Stage 1 – Mild RAO (1 ≤ Total Score < 2);

Stage 2 – Moderate RAO (3 ≤ Total Score ≤ 4);

Stage 3 – Severe RAO (5 ≤ Total Score = 6)
